# Early motor deficits in mouse disease models are reliably uncovered using an automated home-cage wheel-running system: a cross-laboratory validation

**DOI:** 10.1242/dmm.013946

**Published:** 2014-01-13

**Authors:** Silvia Mandillo, Ines Heise, Luciana Garbugino, Glauco P. Tocchini-Valentini, Alessandro Giuliani, Sara Wells, Patrick M. Nolan

**Affiliations:** 1 CNR – Institute of Cell Biology and Neurobiology – EMMA, 00015 Monterotondo Scalo, Italy.; 2 MRC Harwell, Oxfordshire, OX11 0RD, UK.; 3 Istituto Superiore di Sanita, 00100 Rome, Italy.

**Keywords:** Neurodegenerative disease, Complex wheel, Motor function

## Abstract

Deficits in motor function are debilitating features in disorders affecting neurological, neuromuscular and musculoskeletal systems. Although these disorders can vary greatly with respect to age of onset, symptomatic presentation, rate of progression and severity, the study of these disease models in mice is confined to the use of a small number of tests, most commonly the rotarod test. To expand the repertoire of meaningful motor function tests in mice, we tested, optimised and validated an automated home-cage-based running-wheel system, incorporating a conventional wheel with evenly spaced rungs and a complex wheel with particular rungs absent. The system enables automated assessment of motor function without handler interference, which is desirable in longitudinal studies involving continuous monitoring of motor performance. In baseline studies at two test centres, consistently significant differences in performance on both wheels were detectable among four commonly used inbred strains. As further validation, we studied performance in mutant models of progressive neurodegenerative diseases – Huntington’s disease [TgN(HD82Gln)81Dbo; referred to as HD mice] and amyotrophic lateral sclerosis [Tg(SOD1G93A)^dl^1/GurJ; referred to as SOD1 mice] – and in a mutant strain with subtle gait abnormalities, C-Snap25^Bdr^/H (Blind-drunk, *Bdr*). In both models of progressive disease, as with the third mutant, we could reliably and consistently detect specific motor function deficits at ages far earlier than any previously recorded symptoms *in vivo*: 7–8 weeks for the HD mice and 12 weeks for the SOD1 mice. We also conducted longitudinal analysis of rotarod and grip strength performance, for which deficits were still not detectable at 12 weeks and 23 weeks, respectively. Several new parameters of motor behaviour were uncovered using principal component analysis, indicating that the wheel-running assay could record features of motor function that are independent of rotarod performance. This represents a powerful new method to detect motor deficits at pre-symptomatic stages in mouse disease models and should be considered as a valid tool to investigate the efficacy of therapeutic agents.

## INTRODUCTION

A considerable number of human neurological and neuromuscular diseases display elements of motor dysfunction. As more genetic models for these diseases are developed through our increased understanding of their molecular genetic basis, it is incumbent on us not only to map the course of disease progression through the ageing process in these models but also to refine and develop assays that might be used to predict disease onset. Our ultimate goal would be to use discriminatory tests that can identify signatures of motor dysfunction before any debilitating signs of the disease might appear and then follow the course of disease progression through the analysis of the tests’ quantitative measures.

The mouse is amenable to many motor function tests. Because many human disease models have been and continue to be developed in the mouse, mice are vital for determining gene function in disease and in the development of future therapies. Assays for grip strength, placing response, rope grip test, open field locomotor activity, SHIRPA assessment, swim speed, swim quality, gait measurement and rotarod assessment are all widely used to assess motor function in the mouse (for an overview, see Tucci et al., 2006). Moreover, a number of these assays have been used to track disease progression in mouse mutants (Carter et al., 1999; Fernagut et al., 2002; Wooley et al., 2005). A commonly used tool for assessing motor function in rodents is the rotarod test, in which mice or rats are placed on a rotating drum and motor function measured according to the animal’s ability to remain on the drum. Although it is generally accepted that rotarod assessment is useful in any battery of motor function tests, it should not be used alone in assessing motor function in mouse models. There are a number of reasons for this: it can measure only a limited number of motor-related functions, it incorporates a number of confounding coping mechanisms in the mouse that are not strictly motor functions (e.g. mice can cling on and rotate passively as the drum turns), and it can be subject to user-dependent, institute-dependent and equipment-dependent variables (Mandillo et al., 2008; Wahlsten et al., 2003).

These inconsistencies in rotarod test outcomes have prompted investigations into developing more meaningful tests for motor function performance in mice. In recent years, several groups have been assessing new automated methods to measure motor function in mice using automated gait assessment apparatus (Vandeputte et al., 2010; Wooley et al., 2009) or automated horizontal ladder apparatus to record footstep errors (Chort et al., 2013). Although all potentially useful, these new assays are still relatively new and their variability undefined (cf. rotarod testing above). One idea in reducing variability in mouse test outcomes has been to develop assays that can be used for continuous automated home-cage assessment. Historically, wheel running in mice and other rodents has been used as a robust assay to determine, with precision, the inherent period of circadian rhythms in rodents (Bacon et al., 2004). Furthermore, this assay has been instrumental in dissecting the molecular genetic basis of mammalian circadian rhythms. In teasing out the elements of this test that have determined its robustness – automated assessment of voluntary behaviour in the home-cage over long time intervals – we and others have been investigating whether similar test apparatus could be used to determine the onset and progression of motor dysfunction in rodents. Our initial observations using standard wheels, such as those used in circadian rhythms testing, showed that we could identify distinct deficits in running-wheel parameters in dystrophic mice (Hara et al., 2002). At the same time, another group (Schalomon and Wahlsten, 2002) reported the use of a wheel with missing rungs to assess motor function in mice with an absent corpus callosum. Liebetanz and co-workers further refined the method by preceding the complex wheel component with a training period on a standard wheel. With this approach, they were able to detect even subtle differences in mouse models for multiple sclerosis and Parkinson’s disease (Liebetanz et al., 2007; Liebetanz and Merkler, 2006).

**RESOURCE IMPACT**

**Background**

Motor deficits are a major debilitating feature of diverse human disorders, including CNS neurodegenerative diseases, peripheral neuropathies, neuromuscular disorders and musculoskeletal conditions. Considering this diversity, it is surprising that investigations into disease onset and progression in mouse models are confined to the use of a narrow range of tests, including, most notably, the rotarod test. In this test, a rodent is placed on a rotating cylinder and evaluated for its ability to maintain balance and motor coordination. Although considered a useful overall performance test, there are limitations of the rotarod test. Given the recent dramatic rise in the development of new mouse mutant models of neurological disease, it is imperative that additional discriminative tests are developed to investigate motor deficits in these mice as comprehensively as possible.

**Results**

In this study, the authors have developed an automated home-cage version of a challenging wheel-running test for mice. Initial baseline testing was carried out in two European test centres and on four commonly used inbred strains. By analysing multiple distinct parameters in all mice, reliable and robust discriminatory factors delineating motor function were identified. Moreover, using principal component analysis, these factors were found to measure motor capabilities that are distinct from rotarod measures. Finally, by using this apparatus on existing mouse models of neurodegenerative disease and ataxias, early motor deficits could be detected. Deficits were shown to be evident in these mice even at ages that, up to now, have been recorded as pre-symptomatic.

**Implications and future directions**

The new automated wheel-running apparatus reported provides a powerful and discriminative tool for the reliable and reproducible assessment of motor function, as evidenced by its cross-validation across different institutes. The authors demonstrate that the testing apparatus can be used to track the onset and progression of motor function deficits in diverse mouse neurological disease models. Encouragingly, the system can detect early-stage motor deficits that cannot be detected using the rotarod test. Finally, the system will also be of major benefit when conducting longitudinal studies that monitor the long-term effects of therapies.

As part of the EU-funded project PhenoScale, our aim was to develop, refine and automate home-cage wheel running as a method for assessing motor function in mice. During the course of the project, a commercially available version of the standard/complex wheel-running system (PhenoMaster, TSE Systems) was established along with hardware and software for analysing the data obtained. Using pre-determined standard operating procedures (SOPs), the aim of our study was to test reliability, sensitivity, reproducibility and robustness of voluntary wheel running as a measure of motor function in mice. In the first part of this study, we compared baseline test results using four commonly used inbred strains (C57BL/6NTac, C57BL/6J, 129P2/OlaHsd and C3H/HeH, referred to as B6N, B6J, 129 and C3H, respectively) at two test centres (CNR, Monterotondo, Italy and MRC Harwell, UK). This helped us determine whether the motor parameters we were measuring with the system were indeed discriminatory and allowed us to assess the robustness and reproducibility of the data. Furthermore, we determined whether the tests could be used to monitor the onset and progression of motor dysfunction in three mutant strains with distinct aetiologies. The first of these is a progressive model of Huntington’s disease, TgN(HD82Gln)81Dbo (referred to as HD mice). These mice express an N-terminally truncated cDNA containing 82 glutamines and encompassing the first 171 amino acids of huntingtin. These mutant mice develop progressive motor deficits, including loss of coordination, tremors, hypokinesis and abnormal gait, from about 3 months (Schilling et al., 1999). The second mouse line, Tg(SOD1G93A)^dl^1/GurJ (referred to as SOD1 mice), models the human condition of amyotrophic lateral sclerosis (ALS). This widely used mouse model has a lower copy number of human *SOD1* cDNA compared with some other ALS mouse models and represents familial ALS (Gurney, 1994). Upper and lower motor neurons progressively degenerate in this model, leading to paralysis at about 7 months of age (Acevedo-Arozena et al., 2011). Finally, the blind-drunk line (*Bdr*; C-Snap25^Bdr^/H), identified in a large-scale phenotype-driven ENU screen (Nolan et al., 2000), carries a missense mutation in a highly conserved region of SNAP25. Heterozygous mutant mice are grossly normal in both appearance and development, but exhibit, apart from other phenotypes, a subtle abnormal gait pattern that is detectable at about 4 weeks of age. Rotarod tests revealed significantly reduced latencies for *Bdr* animals compared with wild-type (WT) controls and suggested motor function deficits in this mouse line (Jeans et al., 2007).

In developing this new automated wheel-running apparatus, we can report that the system offers a reliable, robust and reproducible test for assessing motor function in mice, as shown by the excellent cross-validation across institutes. The system measures multiple parameters over several weeks in the home-cage and, with any mutant we have tested so far, can detect even early-onset and/or subtle deficits in motor function. It is particularly encouraging to note that this system can be used to detect motor dysfunctions in two widely used models of neurodegenerative disease at stages at which the use of the rotarod has failed to detect any functional deficits.

## RESULTS

### Experimental set up and procedure

**Fig. 1.**
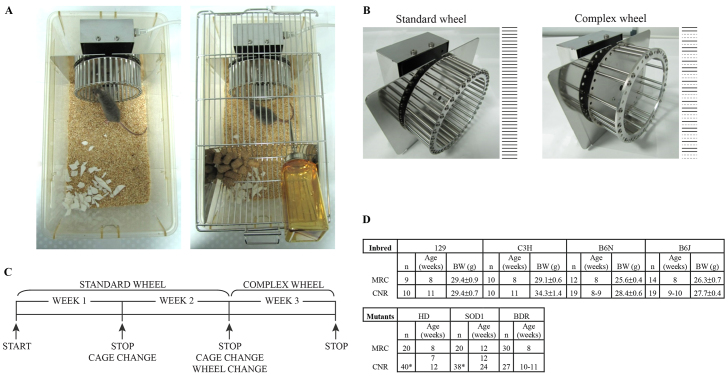
**Wheels and experimental procedure.** (A) Picture of wheel running in home-cage without and with cage lid. (B) Pictures and bar pattern of standard and complex wheels. (C) Schematics of experimental procedure. (D) Table of groups and experimental subjects tested. BW (body weight) mean ± s.e.m. are shown; * two independent groups tested at different ages.

The first aim of the PhenoScale project was to refine and optimise hardware and software for measuring voluntary wheel running activity in mice in their home-cage (Fig. 1A). In doing so, we gave careful consideration to the positioning of running wheels and associated equipment within the cage so as to restrict access to any cables or sensitive equipment. Suitable designs for the complex wheel, for which only half the full complement of rods were present, were investigated and established. We compared a complex wheel missing bars at random positions with one missing bars in distinct alternating patterns and found no differences in running ability. This led us to incorporate the latter complex wheel in this study (Fig. 1B) in addition to the standard wheel containing all rods. Moreover, we established that use of the two patterned complex wheels in series provided no additional valuable information on motor function (result not shown). In order to provide sufficient motivation to run on the more challenging complex wheel, an appropriate training phase before switching from standard wheels was established. We found that animals improved their performance throughout the first week of wheel running, whereas this reached a stable plateau and remained consistent over the second week. So we established a paradigm with 2 weeks training on standard wheels followed by a switch to the complex wheel with an additional week of running (Fig. 1C). During the testing phase mice of both sexes were screened and, because a high variability was evident in female running activity, probably due to the influence of the oestrogen cycle, only male mice were used for further screening in this study (Fig. 1D).

### Standard- and complex-wheel-running pattern in inbred strains

**Fig. 2.**
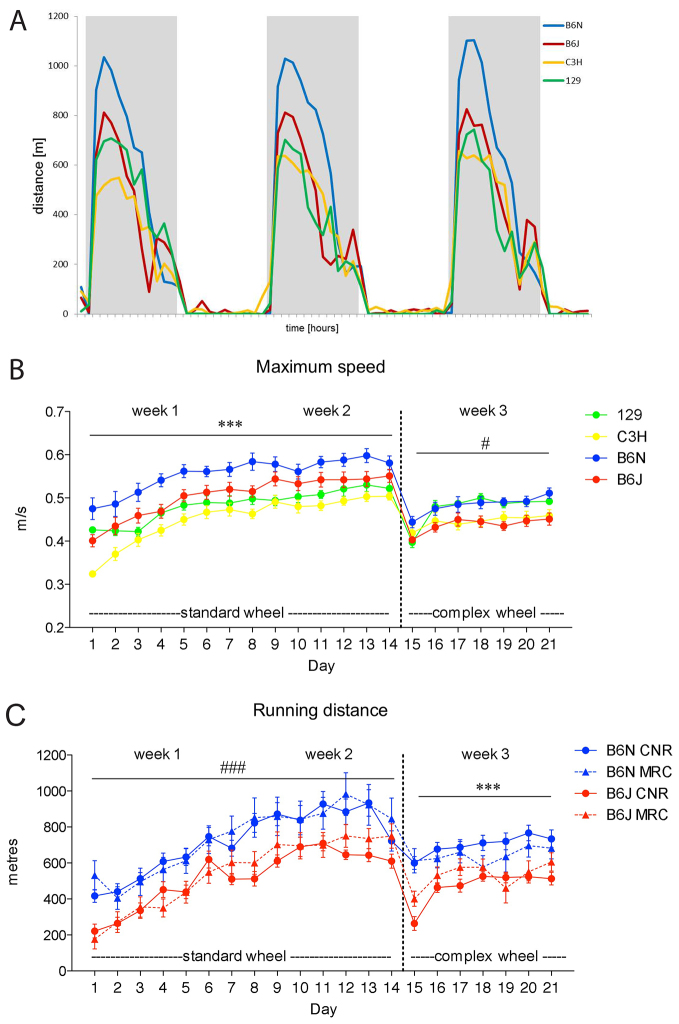
**Running pattern during exposure of inbred strains to standard and complex wheels.** (A) Wheel-running distance (m) and (B) average max speed (m/second) in four inbred strains tested at MRC. In A, white and grey bars correspond to the 12-hour-light and 12-hour-dark phase of the day, respectively. (C) Running distance (m) in B6N and B6J strains tested at CNR and MRC. (B,C) Data (mean ± s.e.m.) are collected from the first hour of daily dark phase along the 3 weeks of access to a running wheel within the home-cage. The dotted line indicates the change from standard to complex wheel. ^#^*P*<0.05, ^###^*P*<0.0005, ***P<0.0001 strain effects. On week 3, *P*<0.0001 day × strain interaction, 129, B6N versus C3H, B6J *P*<0.05 (Fisher’s post-hoc test).

As expected for nocturnal animals, all mouse strains exhibited the vast majority of their wheel-running activity during the dark phase. Consequently, only nightly wheel-running activity was analysed. Although inbred strains showed different wheel-running patterns during the night (Fig. 2A), they all had a very strong onset of wheel-running activity at the beginning of the night starting at lights-off. Initially, we focused on the first hour of nightly activity in determining whether we could detect strain- or genotype-specific effects on motor parameters. However, in considering parameters for a different exploratory statistical approach [principal component analysis (PCA)], we also analysed cumulative nightly activity for weeks one, two and three.

Fig. 2B shows the running-speed pattern during the first hour of the night over the entire 3 weeks of the experiment in one of the test centres (MRC). There are clear quantitative differences in performance among the strains. There is also a clear training effect in all strains, with an increase in average maximum speed during the first week and a more stable activity towards the end of the second week [repeated measures (RM) ANOVA, day effect *F*_(13,468)_=65.434, *P*<0.0001]. B6N mice exhibited the highest speed performance compared with all other strains, and C3H the lowest compared with both B6 substrains [RM ANOVA, strain effect *F*_(3,36)_=12.385, *P*<0.0001].

Voluntary wheel-running activity dropped upon exposure to the complex wheel in all strains. When comparing performance between the last day on the standard wheel and the first day on the complex wheel (transition day effect), average maximum speed was decreased for all inbred strains, reflecting the challenge to run on a wheel lacking bars [RM ANOVA, transition effect: *F*_(1,32)_=150.63, *P*<0.0001, strain effect: *F*_(3,32)_=3.853, *P*=0.018]. More importantly, there were qualitative differences in coping mechanisms in all strains. This was reflected both in a tendency to respond differently to the transition from standard to complex wheel [RM ANOVA, strain × transition interaction: *F*_(3,32)_=2.669, *P*=0.064] and the improvements in wheel running on days subsequent to the wheel change. Interestingly, when switching to the complex wheel, the comparison of running activity showed a different distribution of strain performance compared with that observed with the standard wheel [RM ANOVA week 3, strain effect: *F*_(3,35)_=4.069, *P*=0.014; day effect *F*_(6,210)_=29.225, *P*<0.0001; strain × day interaction: *F*_(18,210)_=2.401, *P*=0.0016]. In particular, 129 and B6N on one hand, and C3H and B6J on the other, showed very similar average maximum speed on the complex wheel, whereas they differed from each other on the standard. These results indicate that the motor ability required to run on the standard wheel might be different from that required to run on the complex wheel.

Fig. 2C compares running distance for B6N and B6J mice at the two research centres (CNR and MRC). The distance travelled on the running wheel was significantly longer for B6N mice compared with B6J during the first 2 weeks (with the standard wheel) [RM ANOVA, *F*_(1,58)_=16.034, *P*=0.0002] and in the week with the complex wheel [RM ANOVA, *F*_(1,58)_=17.545, *P*<0.0001]. Performance of mice was highly comparable between the two centres, demonstrating that this is a readily reproducible paradigm for cross-laboratory validation purposes [RM ANOVA, *F*_(1,58)_=0.247, n.s. and *F*_(1,58)_=3.828E−4, n.s., respectively]. Also, strains coped differently at the transition from standard to complex wheel [RM ANOVA, transition × strain: *F*_(1,58)_=8.933, *P*=0.0041] and during week 3 [RM ANOVA, day × strain *F*_(6,348)_=4.834, *P*<0.0001].

### Standard- and complex-wheel running pattern in mutant models of motor dysfunction

**Fig. 3.**
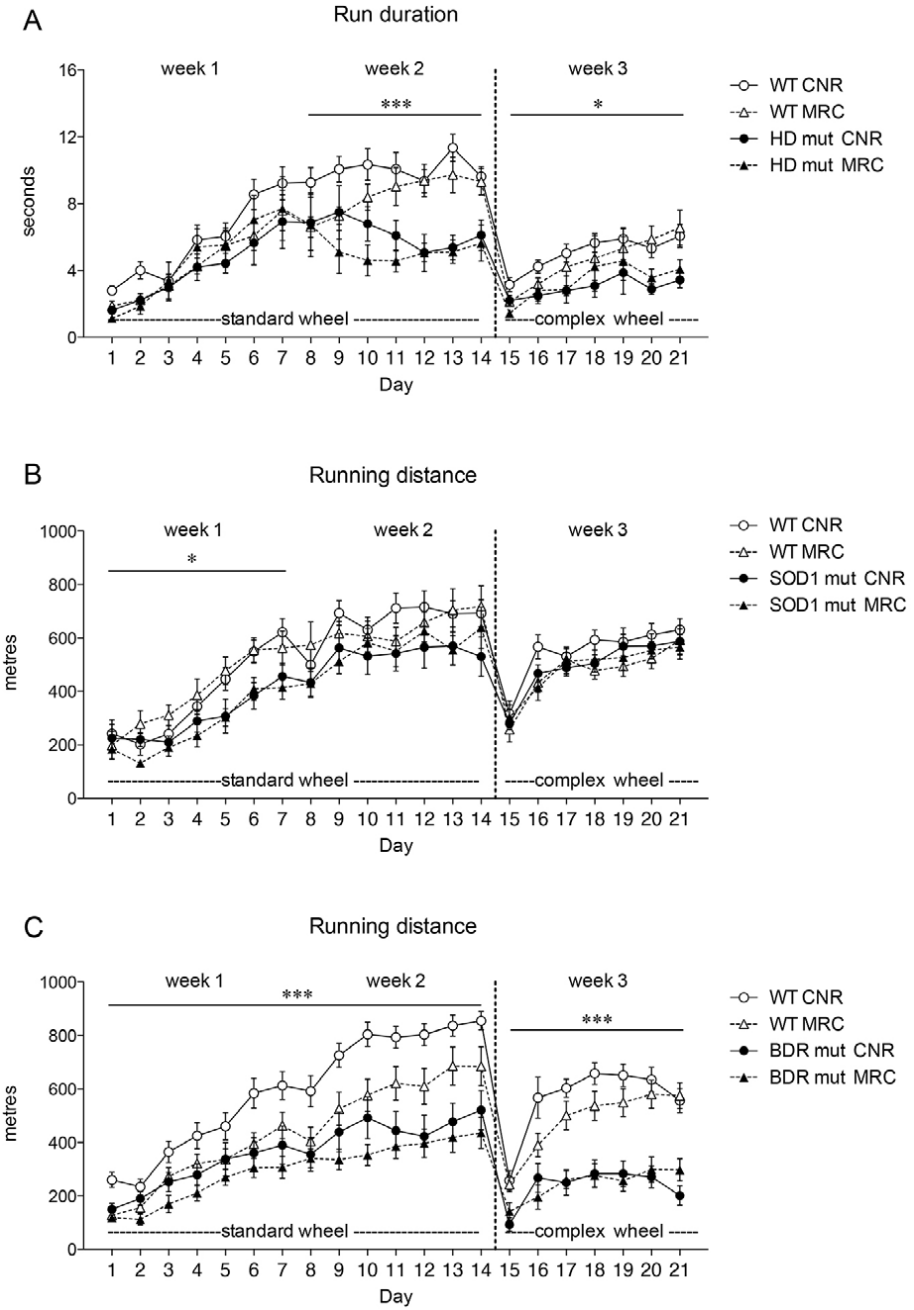
**Running pattern of mutant mice during exposure to standard and complex wheels.** (A) Run duration (seconds) for HD mice (age 7–8 weeks). (B) Running distance (m) for SOD1 mice (age 12 weeks). (C) Running distance for *Bdr* mice (age 8–10 weeks). Data (mean ± s.e.m.) were collected during the first hour of dark phase along the 3 weeks of access to a running wheel within the home-cage at CNR and MRC. *P<0.01; ***P<0.0001 genotype effects; (C) on week 3, *P*<0.0001 day × strain interaction.

Fig. 3 shows examples of wheel running activity during the first hour of the night in three mutant mouse strains with previously recorded motor deficits. The performance of mutants and their littermate controls was compared at the two test centres.

HD animals were tested on the running wheels at 7–8 weeks of age, a stage that had been established as pre-symptomatic in other motor function tests, including rotarod (Corrochano et al., 2012; Ravikumar et al., 2005). The average running duration was significantly reduced in HD mutants compared with WT littermates in both standard and complex wheels (Fig. 3A). Interestingly, the effect of genotype on the standard wheel was observed only during the second week. Here, WT mice continued to increase run duration up to stable levels at the end of the second week, whereas HD mutants did not improve upon their performance in the first week [RM ANOVA first week: *F*_(1,34)_=1.80, n.s. and second week: *F*_(1,35)_=23.65, *P*<0.0001; third week *F*_(1,35)_=8.41, *P*=0.006]. Data also confirmed high reproducibility at the two research centres for both standard and complex wheels [RM ANOVA: *F*_(1,33)_=1.60, n.s. and *F*_(1,35)_=0.005, n.s., respectively]. Similarly, HD mutants also travelled for shorter distances at lower speeds compared with WT mice at both centres (data not shown).

By testing SOD1 animals at the early time point of 12 weeks of age, subtle differences in wheel running behaviour between genotypes were observed during the first week of training (Fig. 3B). Data from the first hour of the night showed reduced running activity in SOD1 mutants during the first week of exposure to the wheel at both testing centres, whereas, during the second week, this difference attenuated, and was no longer detectable during week 3 [RM ANOVA week 1: genotype effect: *F*_(1,32)_=8.902, *P*=0.005; day × genotype interaction: *F*_(6,192)_=3.172, *P*=0.005; centre effect: *F*_(1,32)_=0.043, n.s.; week 2: genotype effect: *F*_(1,33)_=3.439, *P*=0.073; centre effect: *F*_(1,33)_=1.93E−4, n.s; week 3: genotype effect: *F*_(1,31)_=0.334, n.s.; centre effect: *F*_(1,31)_=1.734, n.s.]. These findings could represent an extremely early pre-symptomatic marker for the onset of the disease, because we found additional clear differences in wheel running behaviour in these mice at the later time point of 24 weeks of age (see below).

At both centres, *Bdr* mutant animals had a significantly decreased running distance compared with littermate controls [Fig. 3C; RM ANOVA: first week: *F*_(1,51)_=12.116, *P*=0.001; second week: *F*_(1,51)_=23.969, *P*<0.0001; third week: *F*_(1,50)_=42.943, *P*<0.0001], and also showed a significant difference in average maximum speed and in run duration (data not shown). Distinctively from the other datasets, a difference between centres was observed when mice ran on the standard wheel, but not the complex wheel [first week: *F*_(1,51)_=8.804, *P*=0.005; second week: *F*_(1,51)_=5.589, *P*=0.02; third week: *F*_(1,50)_=0.631, n.s.]. A highly significant genotype × day interaction in week 3 [*F*_(6,300)_=7.45, *P*<0.0001] emphasizes the difference in the ability of mutant mice to cope with the introduction of the complex wheel over time compared with WT. In qualitative terms, wheel running in *Bdr* mice seemed to be less precise and consistent than in WT controls. This indicates a deficit in the ability of *Bdr* animals to coordinate motor functions while running in either standard or complex wheels.

### Robust measures of wheel-running activity using principal component analysis

PCA was performed in order to extract new components of mouse locomotor behaviour when running on the two different wheels. In this comparison, cumulative running activity during the dark phase for each single week was considered for analysis. Parameters included in the PCA were distance travelled, run duration and maximum speed. Thus, a total number of nine variables were included in the PCA. A total of 258 mice, covering all inbred lines and mutants tested, were included in the analysis.

PCA gave rise to a two-component solution accounting for 77.9% of total variance (Table 1). Factor 1 accounted for 66.2% of variance and included almost all variables with very high loadings (>|0.6|), with the only exception being ‘run duration’ during week 3 (complex wheel). This factor has been interpreted as ‘general locomotor activity’ on the wheel. It is worth noting that the parameters most correlated with Factor 1 were those measured in week 2. In fact, mouse running activity stabilized during week 2, as observed in their running pattern during the 3 weeks of exposure to the wheels (Figs 2, 3). ‘Average run duration’ in week 3 was the least correlated with Factor 1, and it is the only variable highly correlated to Factor 2. Factor 2 accounted for 11.7% of total variance and it was interpreted as ‘the ability to run on a complex wheel’. Despite differences in body weights of inbred strain mice (Fig. 1D), no correlation with running-wheel-relevant parameters was observed, i.e. Pearson’s correlation *R* values for body weight versus running distance in week 2 or versus average run duration in week 3 were, respectively, −0.050 and −0.12. We can thus conclude that body weight did not influence wheel running.

**Table 1.**
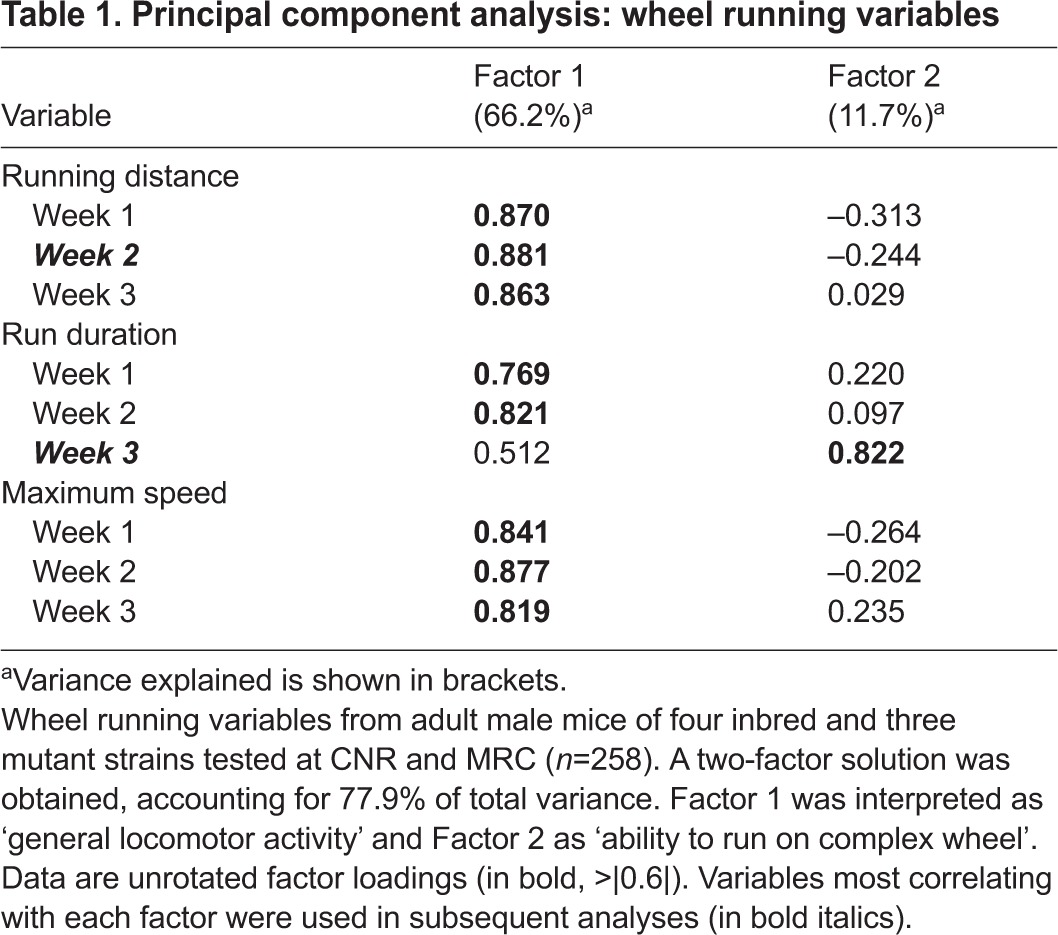
Principal component analysis: wheel running variables

An ANOVA was performed on the two factors, using ‘centre’ and ‘strain’ as independent variables. For Factor 1 (‘locomotor activity’) the ANOVA showed a non-significant effect for both testing centres [*F*_(1,244)_=1.747, *P*=0.188], and for the interaction of centre × strain [*F*_(1,244)_=1.457, *P*=0.194], confirming that wheel-running activity measured at different testing centres is comparable. As expected, we observed a statistically significant effect of strain [*F*_(1,244)_=8.294, *P*<.0001]. For Factor 2 (‘ability to run on complex wheel’) the ANOVA showed a significant main effect of centre [*F*_(1,244)_=4.329, *P*=0.039] and strain [*F*_(1,244)_=2.581, *P*=0.019], but importantly no significant interaction of centre × strain [*F*_(1,244)_=0.486, *P*=0.819].

**Fig. 4.**
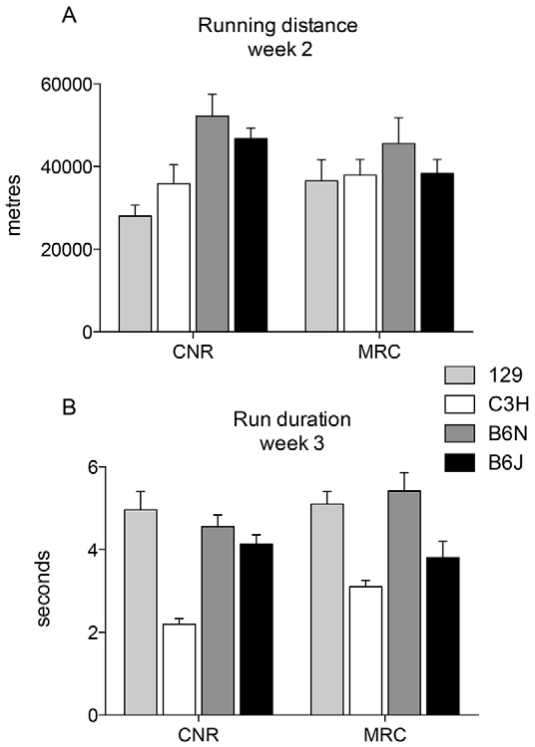
**Cross-validation between centres for inbred mice.** (A) Total running distance in nightly activity of week 2 (standard wheel). 129 versus B6N, B6J, *P*<0.0005 and *P*<0.05, respectively; C3H versus B6N, *P*<0.01 (Fisher’s post-hoc test). (B) Average run duration in night activity of week 3 (complex wheel). C3H versus all *P*<0.0001; B6J versus B6N, 129, *P*<0.005 (Fisher’s post-hoc test). Data are group mean ± s.e.m. collected at CNR and MRC.

To specifically compare running activity among the four inbred strains, evaluate performance in the three mutant mouse lines, and cross-validate results between MRC and CNR, an ANOVA was performed on the running parameters with the highest correlation value within each factor that emerged from the PCA, i.e. ‘running distance week 2’ for Factor 1 and ‘run duration week 3’ for Factor 2. For both parameters, quantitative differences in individual strain performances were evident between centres but strain order of performance was well-conserved (Fig. 4). Moreover, the evidence that two independent motor parameters are being recorded can be seen. Compared with the other inbred strains, despite showing a similar level of locomotor activity on the standard wheel, C3H mice seemed to show a poorer ability to cope with the complex wheel, whereas 129 mice, poor performers on the standard wheel, were among the best performers on the complex wheel. ANOVA confirmed a very high reproducibility between test centres, reporting that differences between centres and the interaction of centre × strain were not significant. Strains differed significantly for both running distance and run duration parameters [running distance week 2, centre effect: *F*_(1,95)_=0.116, n.s.; strain effect: *F*_(3,95)_=4.677, *P*=0.004; centre × strain interaction: *F*_(3,95)_=1.378, n.s.; run duration week 3, centre effect: *F*_(1,84)_=2.71, n.s.; strain effect: *F*_(3,55)_=18.816, *P*<0.0001; centre × strain interaction: *F*_(3,84)_=1.773, n.s.].

**Fig. 5.**
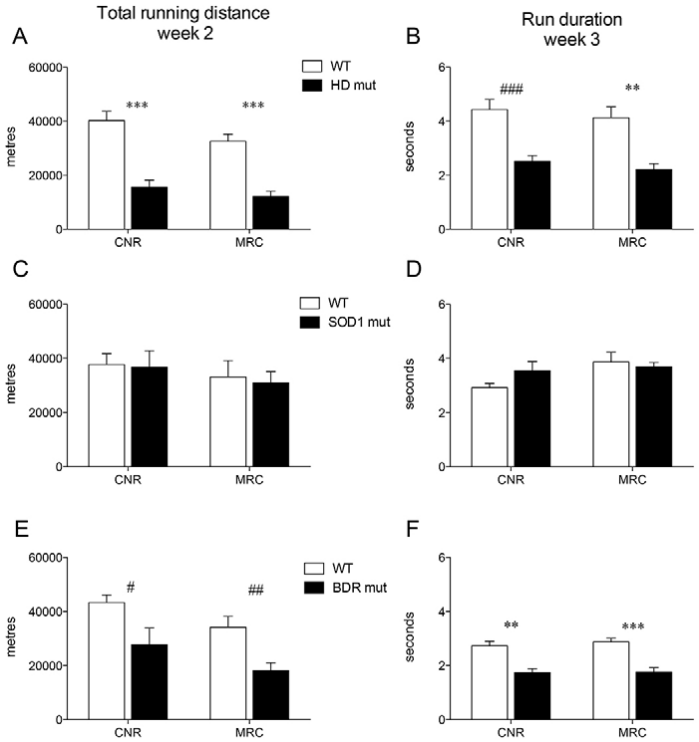
**Cross-validation between centres for mutants.** Total running distance in nightly activity of week 2 (standard wheel), and average run duration in nightly activity of week 3 (complex wheel), in HD (A,B), SOD1 (C,D) and *Bdr* (E,F) mice. ^#^*P*<0.05, ^##^*P*<0.005, ^###^*P*<0.0005, **P<0.001, ***P<0.0001, WT versus mutant (*t*-test). Data are mean ± s.e.m. collected at CNR and MRC.

Both HD and *Bdr* mutant mice showed reduced running distance and poor ability to run on complex wheels compared with their WT littermates (Fig. 5A,B,E,F). However, using the cumulative wheel-running analysis, no differences were observed between WT and SOD1 mutant mice at this time point (Fig. 5C,D), although this was evident at later times. The interaction of centre × strain was never found to be statistically significant in any of the mutant strains, showing that performance was highly comparable between CNR and MRC testing centres. For each mutant strain, an ANOVA was performed on the two selected variables to compare centre and genotype performance (supplementary material Table S1).

### Comparison between running-wheel activity and rotarod performance

In establishing the utility of this test in mouse motor function studies, it was important for us to compare the early-onset wheel-running phenotypes with rotarod performance in the same cohorts of animals. At CNR, running-wheel activity for the three mutant strains was compared with rotarod performance over 4 days (three trials/day). The average latency to fall from the rotarod across the 4 days was included in a separate PCA in addition to the nine variables already described (running distance, average run duration and maximum speed for each of the 3 weeks). In this case, PCA gave rise to a three-component solution accounting for 83.3% of total variance (Table 2). Two components were interpreted as the ones already described in Table 1: Factor 1 (‘general locomotor activity’) accounted for 59.2% of the total variance explained, whereas Factor 3 (‘ability to run on the complex wheel’) accounted for 11% of the total variance explained. A new component emerged, Factor 2 (accounting for 13.1% of the variance explained) that correlated highly (>|0.6|) only with the ‘latency to fall from rotarod’ variable. This factor was therefore interpreted as ‘performance on rotarod’. This result suggests that the home-cage running-wheel and rotarod test measure different and independent motor abilities.

**Table 2.**
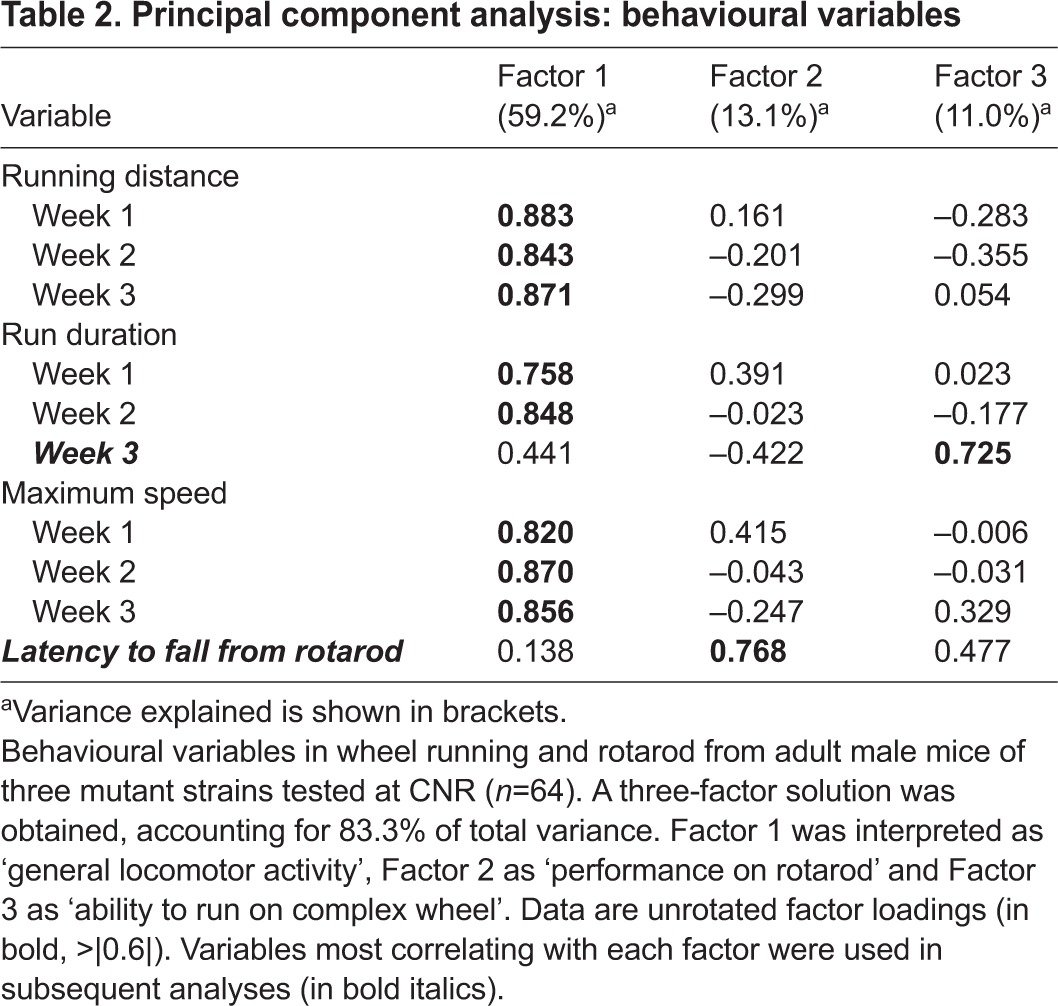
Principal component analysis: behavioural variables

**Fig. 6.**
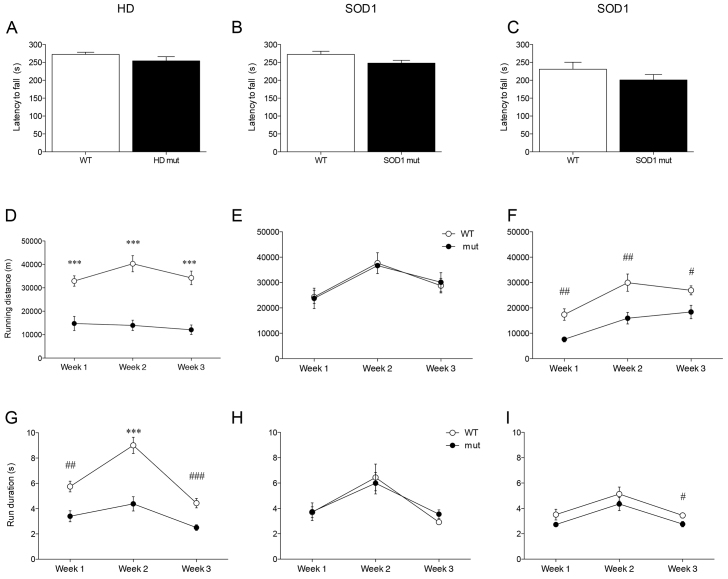
**Comparison between rotarod performance and wheel-running activity in HD and SOD1 mice.** (A–C) Rotarod latency in WT and mutant mice of HD (age 12 weeks) and SOD1 strains (age 17 and 23 weeks, respectively). (D–F) Nightly wheel-running distance and (G–I) average run duration in WT and mutant mice of HD and SOD1 strains running at 7, 12 and 24 weeks of age, respectively. ^#^*P*<0.05, ^##^*P*<0.005, ^###^*P*<0.0005, ***P<0.0001, WT versus mutant. Data are mean ± s.e.m. collected at CNR.

Fig. 6 shows a comparison between rotarod performance and cumulative wheel-running activity in HD and SOD1 mice tested at CNR. No difference in rotarod performance between WT and mutant HD mice was observed at age 12 weeks [*F*_(1,17)_=2.05, n.s., Fig. 6A], nor between WT and SOD1 mice at 17 weeks and even at the later age of 23 weeks [*F*_(1,16)_=1.52, n.s., *F*_(1,18)_=1.473, n.s., respectively; Fig. 6B,C]. In measuring cumulative wheel-running parameters, motor deficits could still be detected in mutants. Compared with WT controls, HD mutants showed reduced running distance and shorter run duration with both standard and complex wheels, even at 7 weeks [ANOVA: distance week 1: *F*_(1,17)_=23.94, *P*=0.0001, week 2: *F*_(1,17)_=39.72, *P*<0.0001, week 3: *F*_(1,17)_=37.38, *P*<0.0001; run duration week 1: *F*_(1,17)_=14.2, *P*=0.001, week 2: *F*_(1,17)_=28.8, *P*<0.0001, week 3: *F*_(1,17)_=18.56, *P*=0.0005; Fig. 6D,G]. With this weekly data analysis, running deficits were not detected in SOD1 mutants at 12–14 weeks [ANOVA: distance week 1: *F*_(1,16)_=0.91, n.s., week 2: *F*_(1,16)_=0.90, n.s., week 3: *F*_(1,16)_=0.79, n.s.; run duration week 1: *F*_(1,16)_=0.96, n.s., week 2: *F*_(1,16)_=0.74, n.s., week 3: *F*_(1,16)_=0.14, n.s.; Fig. 6E,H], but could be detected at 24 weeks, still prior to any detectable rotarod deficit. Overall, running activity was reduced in SOD1 mutants at 24 weeks during the 3 weeks of the experiment [ANOVA: distance week 1: *F*_(1,14)_=15.46, *P*=0.001, week 2: *F*_(1,14)_=11.72, *P*=0.004, week 3: *F*_(1,14)_=7.20, *P*=0.018; Fig. 6F]. In addition, run duration was also reduced in SOD1 mutants, but the difference was statistically significant only during week 3, when animals were exposed to the complex wheel [ANOVA: distance week 1: *F*_(1,14)_=2.96, n.s., week 2: *F*_(1,14)_=1.06, n.s., week 3: *F*_(1,14)_=5.30, *P*=0.037; Fig. 6I].

## DISCUSSION

Reliable measurement of motor function is essential when evaluating rodent models of diseases that are primarily characterized by motor disabilities (Brooks and Dunnett, 2009). In assessing the current status of motor function tests, many researchers would agree that it is imprudent to rely solely on rotarod for motor function assessment, primarily because it does not assess all motor capabilities in rodents. While considering whether new motor function tests can be developed and automated, running-wheel analysis has emerged as a promising candidate. Running wheels are suitable to assess home-cage voluntary activity in an automated fashion and, as in this study, allow the use of a complex wheel to test inbred and mutant mice with a more challenging motor task (Liebetanz et al., 2007; Liebetanz and Merkler, 2006; Schalomon and Wahlsten, 2002). Having developed an automated home-cage version of the paradigm, we can report that the test is reliable and robust, providing highly reproducible data across test centres. In this particular study, we focused on a number of specific parameters that we considered to be most reliable and discriminative based on our validation studies with inbred strains and mutants. Nevertheless, this fully automated system has the potential to investigate many additional parameters that can be procured from the datasets.

As part of the validation process, we have tested performances of a number of inbred strains that are regularly used in mouse studies. In particular, the C57BL/6NTac and 129P2 substrains were specifically chosen in this study because we felt that they would be useful baselines for mouse lines generated in extensive genetargeting studies. 129P2 is the strain used by Baygenomics for their knock-out resource (www.mmrrc.org) and C57BL/6NTac is used for the International Mouse Phenotyping Consortium (IMPC; www.mousephenotype.org). Extensive phenotyping data for each (including growth curves, rotarod and open field) is available at www.europhenome.org.

All mice reached stable running during the second week of continuous exposure to a standard wheel, showing a typical training curve. On the third week, upon changing the wheel from standard to complex, we found a sharp decrease in the performance of all the strains tested, demonstrating a strong effect of the motor challenge that they were exposed to. As previously shown, running on an irregular wheel requires higher running abilities, and could be a valid tool to identify latent and/or subtle motor deficits not detectable with the standard wheel (Liebetanz et al., 2007; Liebetanz and Merkler, 2006; Schalomon and Wahlsten, 2002).

Inbred strains showed marked differences in wheel-running performance and, more interestingly, these differences depended on the type of wheel being used. B6N mice showed the highest performance compared with all other strains. This is in contrast to B6J versus B6N locomotor parameters in other behavioural tests, including open field, water maze and rotarod tests (Simon et al., 2013), in which B6N mice perform less well. Paradoxically, 129 mice showed low performance on the standard wheel (Fig. 2B; Fig. 4A), but they seem to adjust to the complex wheel just as easily as B6N mice (Fig. 2B; Fig. 5B). The different impact of the complex wheel among strains is strikingly evident also in C3H mice, who, despite having similar levels of total running distance on a standard wheel, showed lower run duration during week 3 compared with the other strains. All of these data confirm that complex-wheel running measures motor abilities that are independent of general locomotion.

HD and *Bdr* mutants showed a markedly lower running performance compared with WT with both the standard and complex wheel. Our findings show a distinct and reproducible motor deficit at 7–8 weeks of age in HD mice, preceding any published deficit for this particular mutant by ~4 weeks (Corrochano et al., 2012; Ravikumar et al., 2005; Schilling et al., 1999). The age of onset of this particular deficit could not be tested because the current system is not appropriate for use in younger animals. The complex wheel data gave no additional information on HD mutant performance, although this was undoubtedly compromised during the third week of testing. It is possible that this is characteristic of mice with particular and specific motor disabilities. In contrast, although *Bdr* mice were also compromised in standard-wheel performance like HD mice, we saw additional specific deficits in complex-wheel performance. This could indicate that *Bdr* mice are generally deficient in a spectrum of motor function modalities because they have also been found to have deficits in rotarod tests with a subtle gait anomaly from weaning (Jeans et al., 2007). Finally, SOD1 mutants show a subtle deficit in running activity at 12 weeks that is not detectable when analysing cumulative wheel-running performance at this time. This subtle deficit is detected ~3 months before the earliest published motor phenotype in these mice (Acevedo-Arozena et al., 2011; Gurney, 1994) and only emerged when analysing running activity at the beginning of the dark phase during the first week. The subsequent disappearance of this effect during weeks 2 and 3 could be due either to an increase in motivation in mutant mice or an effect of training, which ameliorates running performance over time. Nevertheless, wheel-running effects at 12 weeks were not an anomaly because additional cumulative wheel-running deficits were clearly detected in SOD1 mice at 24 weeks of age.

### Assessment of motor function using running wheels or rotarod

As one of the most widely used tests to assess the onset of motor symptoms in rodent models of neurodegenerative diseases such as HD and ALS (Carter et al., 1999; Dunham and Miya, 1957; Weydt et al., 2003), the rotarod test has been evaluated repeatedly in terms of reliability and reproducibility of test results between different labs. In general it emerged as a test that is highly sensitive to experimental and environmental variables. In a prominent study it was even concluded that the rotarod data were essentially uninterpretable and thus were not presented (Crabbe et al., 1999). In another study it was possible to attain a good level of comparability across three test centres but this was achieved only after careful redefinition of the standard procedure and modification of the material covering the rotating rod (Mandillo et al., 2008). Conversely, the results from our study would argue for the widespread use of a wheel-running test in studying progressive motor deficits in mice. The test is robust, invariable across test centres, carries a low risk of data misinterpretation, and, most significantly, improves the likelihood of detecting the early onset and progression of motor function deficits in disease models.

A significant finding from the PCA is that running on the complex wheel seems to require a specific motor ability that is independent from general locomotion, and from skills required on the rotarod. The latency to fall from the rotarod correlates with a factor in the PCA that is independent from run duration on the complex wheel or from any other general activity parameter on a standard wheel. Liebetanz and Merkler already proposed the complex wheel as a sensitive tool to assess motor impairment that could be an alternative to the rotarod, balance beam and grip strength test, and they emphasized the reliability of the complex wheel in detecting latent motor deficits in their multiple sclerosis mouse model (Liebetanz and Merkler, 2006).

Here, we showed different wheel-running abilities in inbred strains that do not correspond with rotarod performance. Namely, C3H had a very low run duration on the complex wheel, whereas their performance on rotarod has been reported to be better than other strains (Mandillo et al., 2008). Similarly, B6N were the strongest performers in this apparatus yet were shown in a recent study to perform worse than B6J on the rotarod and are deficient in locomotor parameters in other behavioural tests, including open field and water maze tests (Simon et al., 2013). Additionally, both HD and SOD1 mutant mice displayed motor deficits with the running wheels that were undetectable during rotarod testing. These findings suggest that the two instruments are measuring different motor skills (e.g. ability versus coordination) that could be reflecting the differential involvement of distinct brain regions, e.g. cerebellum, corpus callosum, striatum, motor cortex, medulla and spinal cord (Penhune and Steele, 2012).

### Conclusions

We have developed a new automated wheel-running system that provides reliable, robust and reproducible phenotyping data for the evaluation of specific motor functions in mice. This system can measure home-cage voluntary running behaviour automatically over several weeks. The standard procedure and the apparatus have been successfully cross-validated in two research centres using inbred strains as well as in two widely used mouse models of neurodegenerative disease. We have demonstrated that the system is sensitive enough to detect motor deficits in mutants at pre-symptomatic stages, whereas the rotarod did not show any impairment. This system proves to be a reliable and sensitive method for the detection of distinct motor deficits in mouse models of disease and will be a major benefit in longitudinal therapeutic studies. Also, given the automated home-cage voluntary nature of the test it is a straightforward and welcome addition to the existing battery of motor function tests.

## MATERIALS AND METHODS

### Animals and husbandry

Adult male mice of four inbred strains – 129P2/OlaHsd (129), C3H/HeH (C3H), C57BL/6NTac (B6N) and C57BL/6J (B6J) – and of three mutant strains – B6-TgN(HD82Gln)81Dbo/H (HD), Tg(SOD1G93A)^dl^1/GurJ (SOD1) and C3;C-Snap25^Bdr^/H Blind-drunk (*Bdr*) – were tested at two different research centres: Medical Research Council (MRC) – Mammalian Genetics Unit, Harwell, UK, and National Research Council (CNR) – Institute of Cell Biology and Neurobiology, Monterotondo, Italy.

Both transgenic lines were backcrossed for at least 12 generations to C57BL/6J; heterozygous C3;C-Snap25^Bdr^/H mice were kept on a C3H/HeH background. For mutant strains, WT littermates served as controls. Inbred and mutant mice tested at MRC were all bred in-house, and mice tested at CNR were obtained from MRC, with the exception of C57BL/6J and C57BL/6NTac mice, which were bred at CNR-EMMA facility (Monterotondo, Italy).

At MRC, mice were group-housed in IVC cages (Techniplast, London, UK) enriched with shredded tissue and cardboard funnel tunnels (Datesand, Manchester, UK). Food (SDS RM3 E – expanded diet, Witham, Essex, UK) and water were available *ad libitum*. Room temperature was 21±2°C, relative humidity was 45–65% and mice were kept in a 12-hour light/dark cycle with lights on at 7 am. At CNR, mice were group-housed in standard cages (Thoren, Hazleton, PA) enriched with a transparent red polycarbonate igloo house (Datesand, Manchester, UK) and with wood shavings contained in single cellulose bags (Scobis Uno bags, Mucedola, Settimo Milanese, Italy). Food (2918 Teklad diet, Mucedola, Settimo Milanese, Italy) and water were available *ad libitum*. Room temperature was 21±2°C, relative humidity was 50–60% and mice were kept in a 12-hour light/dark cycle with lights on at 8 am.

At MRC, animal studies described in this paper were carried out under the guidance issued by the Medical Research Council in ‘Responsibility in the Use of Animals for Medical Research’ (July 1993) and Home Office Project Licence No. 30/2686. All experiments conformed to international guidelines on the ethical use of animals. At CNR, animals were subjected to experimental protocols approved by the Veterinary Department of the Italian Ministry of Health, and experiments were conducted according to the ethical and safety rules and guidelines for the use of animals in biomedical research provided by the relevant Italian laws and European Union directives (n. 86/609/EEC and subsequent).

### Wheel-running activity

Running activity was observed in a total of *n*=278 mice (for number and age of animals see Fig. 1D). Mice were single-housed and a running wheel (TSE Systems, Bad Homburg, Germany) was placed in each home-cage during the experiment. All the equipment and TSE PhenoMaster software are commercially available at TSE Systems (http://www.tse-systems.com/). In our study we used 20 wheel-running cages simultaneously, but the TSE Running Wheel System can support up to 32 cages. All training in hardware and software management and data analysis is straightforward. It requires basic handling skills with mice and some practice in assembling and disassembling the running wheels. Although the handling time and the investigator time required during the experiment is little and mainly devoted to checking the functioning of the wheels, a significant amount of time is necessary for data handling, organizing and analysis. Data of three consecutive weeks of wheel-running activity were automatically collected and processed with PhenoMaster software (TSE Systems). In week 1 and week 2, mice were exposed to a standard wheel, whereas, during week 3, a ‘complex’ wheel was introduced. This wheel has fewer rods and they are irregularly spaced, although with a predictable pattern (Fig. 1B) (Liebetanz and Merkler, 2006). Food and water were available *ad libitum*, and nesting was provided. At CNR, running wheels were placed in standard cages (Type II long, Ebeco, Germany) in a dedicated room with the same light/dark cycle and environmental conditions as in the housing room. At MRC, standard cages were equipped with a running wheel and kept in a ventilated cabinet (Scantainer^CLASSIC^ Z-11E, Scanbur-Technology).

Wheel-running activity either for the 12-hour dark phase or the first hour in darkness was included in this study. Parameters measured were weekly values of: (1) total distance (m); (2) average run duration (seconds), where ‘runs’ are running episodes at a velocity exceeding 30 rpm (~0.18 m/second); (3) average maximum speed (m/second) from daily values calculated as average of maximum speed values sampled every 5 minutes during the 12-hour dark period.

### Rotarod

At CNR, HD and SOD1 male mice were also tested on the rotarod (Ugo Basile, Como, Italy). HD mice (*n*=10 WT, 9 Tg) were tested at 12 weeks of age, and SOD1 mice (*n*=8 WT, 10 Tg) were tested at 17 weeks, after the 3-week wheel-running experiment. A different cohort of SOD1 mice (*n*=10 WT, 10 Tg) was tested at 23 weeks of age. Mice had to keep their balance on a rotating rod (3-cm diameter) set at an accelerating speed from 4 to 40 rpm in 300 seconds (mod. 47600 apparatus; Ugo Basile, Como, Italy). To familiarize with the apparatus, mice underwent a training session of three trials, 60 seconds each, in which the rod was kept stationary for the first trial and held at 4 rpm for the last two trials. The next day, for 4 consecutive days, mice were tested over three trials/day with an inter-trial interval of approx. 30 minutes. A maximum of three mice were placed on the rod at the same time. The latency to fall from the rotating rod was recorded in each trial. If a mouse was passively rotating on the rod (i.e. clinging) the number of passive rotations were counted. For each day data were expressed as mean latency to fall minus a 1-second ‘penalty’ for each passive rotation. Mean 4-day latency was considered for analysis.

### Statistical analysis

A repeated-measures ANOVA (RM ANOVA) was performed on most datasets with STRAIN or GENOTYPE and CENTRE as between-subject factors and DAY as within-subject factor. Fisher’s post-hoc tests were performed when possible.

A factor analysis of wheel-running activity using a principal component analysis (PCA) with unrotated factors was performed including all animals, except aged (24 weeks) SOD1 mice (total *n*=258). Nine variables were included in the PCA: distance, run duration and maximum speed, calculated for each single week of measurement. A two-way ANOVA with CENTRE and STRAIN as main factors was performed to analyse the components extracted with PCA. Two-way ANOVAs with CENTRE and STRAIN, and CENTRE and GENOTYPE were also separately performed in inbred and mutant strains for cross-validation analysis of running parameters. Unpaired *t*-tests comparing inbred strains within each centre and between centres were performed.

A separate PCA was also performed including wheel-running and rotarod variables (total ten variables). Rotarod performance in each mutant strain was analysed through a one-way ANOVA with GENOTYPE as main factor. Post-hoc analysis was performed where possible. Subjects whose individual values differed ± 2 s.d. from the group average were considered outliers and were excluded from analysis. Most of the exclusions were due to technical issues. Significance level was set at *P*<0.05. Data are presented as mean ± s.e.m. All statistics were run using the StatView 5.0 PowerPC (SAS Institute Inc., Cary, NC) software package.

## Supplementary Material

Supplementary Material
