# Supplementary material for: Early motor deficits in mouse disease models are reliably uncovered using an automated home-cage wheel-running system: a cross-laboratory validation
Source: Dis Model Mech. 2014 Jan 13;7(3):397–407. doi: 10.1242/dmm.013946 (PMC3944499; doi:10.1242/dmm.013946)
Supplement: Supplementary Material [file supp_7_3_397__index.html]

Early motor deficits in mouse disease models are reliably uncovered using an automated home-cage wheel-running system: a cross-laboratory validation — Supplementary Material 

# Early motor deficits in mouse disease models are reliably uncovered using an automated home-cage wheel-running system: a cross-laboratory validation

## DMM013946 Supplementary Material

**Files in this Data Supplement:**

- **Supplementary Material**
