# Supplementary material for: Early motor deficits in mouse disease models are reliably uncovered using an automated home-cage wheel-running system: a cross-laboratory validation
Source: Dis Model Mech. 2014 Jan 13;7(3):397–407. doi: 10.1242/dmm.013946 (PMC3944499; doi:10.1242/dmm.013946)
Supplement: Supplementary Material [file supp_7.3.397_DMM013946.pdf]

Table S1

			Centre		Genotype		Centre x Genotype	
		DF	F	p	F	p	F	p
HD	Distance week 2	1, 35	4.142	0.0495	67.683	<0.0001	0.594	0.4462
	Run dur. week 3	1, 35	0.93	0.3414	36.333	<0.0001	5.876E-5	0.9939
SOD1	Distance week 2	1,33	0.951	0.3365	0.088	0.7684	0.013	0.9107
	Run dur. week 3	1,32	3.751	0.0617	0.643	0.4284	2.02	0.1649
BDR	Distance week 2	1,48	5.905	0.189	16.631	0.0002	0.004	0.9497
	Run dur. week 3	1,48	0.243	0.624	41.012	<0.0001	0.132	0.7177

ANOVA results of mutant strains cross-validation.
